# Microstructure-Enhanced Liquid–Liquid Extraction in a Real-Time Fluorescence Detection Microfluidic Chip ^†^

**DOI:** 10.3390/mi7030046

**Published:** 2016-03-10

**Authors:** Penghui Xiong, Xiangyu Chen, Ying Xiong, Gang Liu, Yangchao Tian

**Affiliations:** 1 Department of Precision Machinery and Precision Instrumentation, University of Science and Technology of China, Hefei 230026, China; xph@mail.ustc.edu.cn (P.X.); cxy0910@mail.ustc.edu.cn (X.C.); 2 National Synchrotron Radiation Laboratory, University of Science and Technology of China, Hefei 230029, China; xywch@ustc.edu.cn

**Keywords:** microfluidic chip, liquid–liquid extraction, lithography, fluorescence detection

## Abstract

Microfluidic system is widely employed in the detection of environmental contaminants and biological specimens. One of the critical issues which limits the applications of microfluidic chips is the limit of detection of trace specimens. Liquid–liquid extraction is of great importance in the preprocessing in microfluidic devices. In this paper, we developed a real-time fluorescence detection microfluidic chip combined with a microstructure-enhanced liquid–liquid laminar extraction technique, which concentrated the trace compound and realized real-time monitoring. Auxiliary microstructures integrated in the microfluidic chip were applied to increase the extraction efficiency, which was proved by the FEM (finite element method) simulation as well. A common fluorescence probe, Rhodamine 6G (Rh6g), was used in the experiment to demonstrate the performance of the microfluidic system. It revealed that the liquid–liquid laminar extraction combined with auxiliary microstructures of a cross shape was an effective method for enrichment. The efficiency of microstructure-enhanced liquid–liquid extraction was increased by 350% compared to the traditional laminar flow extraction.

## 1. Introduction

Micro total analysis system (μTAS), which is called “lab-on-a-chip,” has developed rapidly in past decades [1,2,3,4]. Various microfluidic devices have been manufactured, such as material synthesis [5], chemical analysis [6], and cell manipulation [7]. Moreover, lab-on-a-chip devices take advantage of various detection strategies to achieve micro volume detection, such as high performance liquid chromatography [8], surface-enhanced Raman spectroscopy [9,10], *etc.* Additionally, laser-induced fluorescence spectroscopy has received great attention as potential applications for environmental monitoring [11] due to its easy coupling, rapid response, low solvent consumption, and high sensitivity. However, it is complex for the preprocessing of specimens and the subsequent monitoring operation, and it takes up the vast majority of labor and time in the whole process of analysis [12].

Great efforts have been applied to the preprocessing in microfluidic devices. Diffusion-based extraction was applied to separate particles and molecules by transferring them from one fluid to another [13,14]. However, it is not suitable to separate particles with similar sizes or diffusion coefficients. Furthermore, two-phase laminar extraction was applied to separate or concentrate targeted chemical compounds or analytes in industrial and laboratory areas [15,16,17]. Through the concentration and separation of analytes, the limit of detection was improved fundamentally. In the aforementioned microfluidic devices, the stable laminar flow is the most common fluid status, which is formed between aqueous and organic phase solutions. There are still several devices that are insufficient in stable liquid–liquid laminar extraction. The enrichment factor was affected by the solvent concentration and distribution ratio of the two phases. At low Reynolds numbers, the extraction efficiency is limited by the slow interfacial exchange rate [17]. For that reason, how to increase the extraction efficiency in the microfluidic chip deserves a further comprehensive study. Promoting the mixture in each phase and improving mass transfer in the interface were considered to be effective solutions. Maruyama *et al*. proposed a method to promote solvent extraction of metal ions in a microfluidic device through the use of intermittent partition walls [18]. The periodic partition walls induced turbulence in the two-phase flow, promoting transport of the metal ions. The periodic partition walls served as guidelines rather than promoters of the laminar extraction with limited improvement of extraction efficiency. Marschewski *et al*. presented herringbone-inspired microstructures in co-laminar microfluidic devices [19]. The reaction and extraction were enhanced by promoting convective mixing in each reactant region to improve mass transfer to the reactive boundaries.

In this study, we designed and manufactured a real-time fluorescence detection microfluidic chip, integrated with the advantages of fluorescence detection and liquid–liquid extraction. Auxiliary microstructures of a cross shape were designed in the microfluidic chip to improve the laminar flow extraction, with the purpose to introduce a slight turbulence after the bulge of the cross shape and keep a steady laminar flow in the whole channel, which were proved by the finite element method (FEM) simulation. The proposed microfluidic chip with auxiliary microstructures was put into use and its performance was demonstrated by common fluorescence probe, Rhodamine 6G. The auxiliary microstructure of the cross shape was demonstrated effectively by accelerating the enrichment of preconcentration prior to fluorescence detection. The extraction efficiency of the microstructure-enhanced microfluidic system was increased by 350% compared to the traditional laminar flow extraction. The microfluidic device provided a quick and *in situ* detection method with great potential in the application of environmental monitoring and biological detection.

## 2. Materials and Methods

### 2.1. Chemicals and Apparatus

All of the reagents used were analytically pure grade, and pure water was used throughout the experiment. An aqueous stock solution of Rhodamine 6G (Rh6g) was prepared by dissolving Rh6g in pure water and sequentially diluting the stock solution with water into variable concentrations. Meanwhile, the standard organic stock solution of Rh6g was prepared by dissolving in n-octyl alcohol and sequentially diluting the stock solution with n-octyl alcohol into variable concentrations.

The microfluidic chip device, equipped with a syringe pump (LSP01-1A, Baoding Longer Precision Pump., Ltd., Baoding, China) and a peristaltic pump (BT100-2J, Baoding Longer Precision Pump., Ltd.) via silastic tube, was used for liquid–liquid laminar extraction. A 532-nm green laser (MLL-III-532, Changchun New Industries Optoelectronics Tech. Co., Ltd., Changchun, China) and a fiber optic spectrometer (USB2000+, Ocean Optics, Dunedin, FL, USA) was coupled into the microchip device with multimode optical fibers, which were applied for real-time fluorescence detection.

### 2.2. Design and Fabrication

Three kinds of auxiliary microstructures were designed to promote the liquid–liquid extraction—a blank channel, a rectangle shape, and a cross shape. The first two cases were frequently used in microfluidic devices [18,20]. The auxiliary microstructure of the rectangle shape serves as guidelines rather than promoters of extraction. The cross shape was designed to improve the extraction efficiency by introducing a slight turbulence after the bulge of the cross shape [21]. The auxiliary microstructures were assembled along the center line of the microchannel with a period of 2 mm spread across the channel with the length of 30 mm and the width of 600 μm. The microstructure of the cross shape was 50 μm wide and 1 mm long. In order to avoid jams in the microchannel and form sufficient perturbation in the microfluidic chip, the bulge of the cross shape was 70 μm wide and 150 μm long after comprehensive consideration, perpendicular in the middle of the long strip.

**Figure 1:**
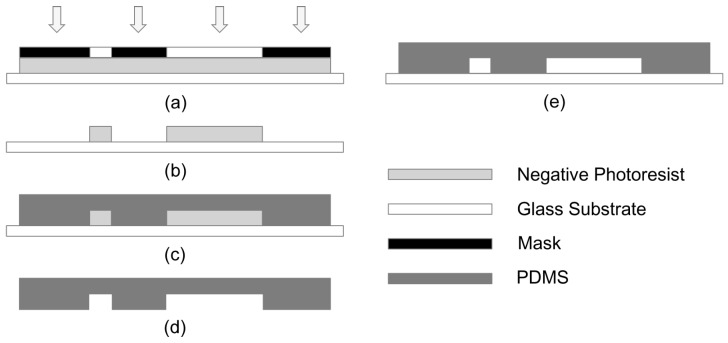
The scheme of the microfluidic chip fabrication: (**a**) photolithography process; (**b**) developing process; (**c**) cast process; (**d**) PDMS demolding process; (**e**) bonding process.

Photolithography was performed to pattern microchannels onto an 80 mm × 80 mm × 1.7 mm glass substrate with SU-8 2100 negative photoresist (MicroChem Corp., Westborough, MA, USA) with a height of 125 μm. Polydimethylsiloxane (PDMS, SYLGARD 184 Silicone Elastomer Kit, Dow Corning, Midland, MI, USA) molding was used for duplicating the microchannels and bonding the PDMS microchannels onto a 1.7-mm-thick 20 mm × 70 mm glass slide [22]. The schematic diagram of the microfluidic chip fabricating procedures is illustrated in Figure 1. In detail, the glass substrate was sliced into squares (80 mm × 80 mm), washed with ultrasonic detergent in acetone for 4 min, rinsed in deionized (DI) water step by step, and dried on the heated platen at 120 °C for 30 min immediately. The 125-μm-thick negative photoresist of SU-8 2100 was then spin-coated on the glass substrate. After photolithography and developing processes as shown in Figure 1a,b, a thick layer of PDMS (3 mm) was cast on the patterned glass substrate subsequently. The PDMS was a two-part system with a mix ratio of cross-linker/curing agent A:siloxane B = 1:10. Upon manually stirring with a glass rod, the PDMS was degassed in a vacuum oven afterwards. An appropriate amount of PDMS was poured on the patterned glass substrate, as shown in Figure 1c. Then, a 24-h curing process was operated by placing the PDMS and photoresist in an oven at 50 °C. After gradually cooling to ambient temperature, the PDMS mold could be easily detached from SU-8 2100 microchannels without resistance, as shown in Figure 1d. Thereafter, after edge modifying and ultrasonic cleaning in acetone for 4 min, the PDMS replica and the 1.7-mm-thick 20 mm × 70 mm glass slide were surface-treated by O_2_ plasma. Immediately, the PDMS was pressed against the pretreated glass substrate. A self-made clamp was used to fasten and force the PDMS and glass slide to adhere to each other closely, as shown in Figure 1e. After 1 h of thermal compression bonding process in an oven at 90 °C and gradually cooling to ambient temperature, a real-time detection microfluidic chip was fabricated [23].

### 2.3. Procedures

**Figure 2:**
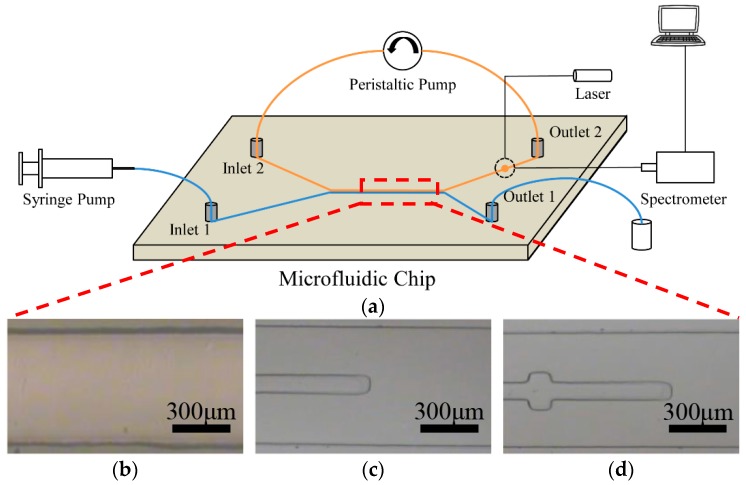
(**a**) An operation schematic diagram of the real-time fluorescence microfluidic chip (blue lines: aqueous phase; orange lines: organic phase), (**b**–**d**) the microphotograph of auxiliary microstructures lay in the center line of microchannels: the blank channel, the rectangle shape, the cross shape respectively.

The laser-induced fluorescence detection was performed using a laser source and a fiber optic spectrometer (USB2000+, Ocean Optics, Dunedin, FL, USA). As shown in Figure 2a, the testing Rh6g solvent was pumped into the PDMS microfluidic chip via a syringe pump continuously from inlet 1. Cycling extraction was realized by a peristaltic pump, connecting inlet 2 and outlet 2 together. Owing to the low Reynolds numbers, a stable laminar flow of aqueous phase and organic phase turned up in the microchannel. The Rh6g molecule was extracted from the aqueous phase into the organic phase on the interface of two phases. After the extraction circulation of about half an hour, the fluorescence detection of the organic phase containing a high concentration of the Rh6g molecule was carried out in a liquid pool prior to outlet 2. The microfluidic chip coupled with the laser source and fiber optic spectrometer by multimode optical fibers. The optical fibers were inserted into the PDMS microfluidic chips by reserved channels, which were fabricated in the microfluidic chip duplicated simultaneously. In order to reduce the interference of exciting light, the emission fiber was set up perpendicular to the excitation fiber [16].

## 3. Results and Discussion

### 3.1. Selection of Flow Rate

**Figure 3:**
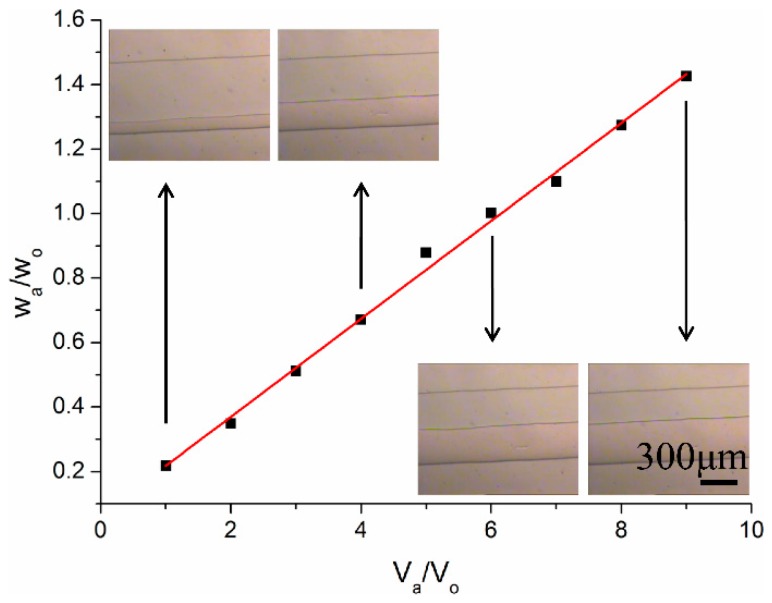
The standard curve of the relationship between the flow width ratio and the velocity ratio (inset: the microphotograph of stable laminar flow corresponding to individual velocity ratio, the top phase was the aqueous phase of pure water and the bottom phase was the organic phase of n-octyl alcohol).

A stable laminar flow extraction came to being in the middle of the channel of a hollow microfluidic chip. For the sake of comparison, the condition of two-phase must be controlled carefully. Theoretically, the ratio of width is inversely proportional to the viscosity of two phases [24]. The larger the viscosity, the narrower the phase flow is. The standard curve of the relationship between the flow width ratio and the velocity ratio was carried out as shown in Figure 3. The velocity of organic phase was fixed at 0.1 mL·h^−1^, and the velocity of the aqueous phase was set from 0.1 to 0.9 mL·h^−1^. The image of stable laminar flow was observed under the microscope (Nikon Optiphot 100, Tokyo, Japan), as shown in the inset of Figure 3. The top phase was the aqueous phase of pure water, and the bottom phase was the organic phase of n-octyl alcohol. The slope of the fitted curve was calculated as 0.152. The equation of the relationship between the flow width ratio and the velocity ratio could be expressed as:

(1) $$\frac{w_{a}}{w_{o}}≅0.152\times \frac{v_{a}}{v_{o}}$$

where *w_a_* and *w_o_* is the width of the aqueous phase and the organic phase, respectively, and *v_a_* and *v_o_* the velocity of the aqueous phase and the organic phase, respectively.

A stable laminar flow came up in the hollow microchannel of microchip device. In order to separate the two phases at the end-junction of the microchannel conveniently, the flow rate was set to be half apart empirically [16]. Furthermore, if the width of the aqueous phase was larger than that of the organic phase, the solvent far from the two-phase interface had nothing to do with the extraction. More feed solvent was needed. Similarly, if the width of the aqueous phase was narrower than the organic phase, it was difficult to acquire a high concentration in the organic phase. To obtain the same enrichment ratio, more extraction cycles and efforts were needed. Hence, the half-and-half flow state was controlled in the laminar extraction. In order to maintain the stable half-and-half divided flow state, the value of *v_a_*/*v_o_* should be limited to approximately 6 for convenience.

### 3.2. Simulation of Flow Flied

**Figure 4:**
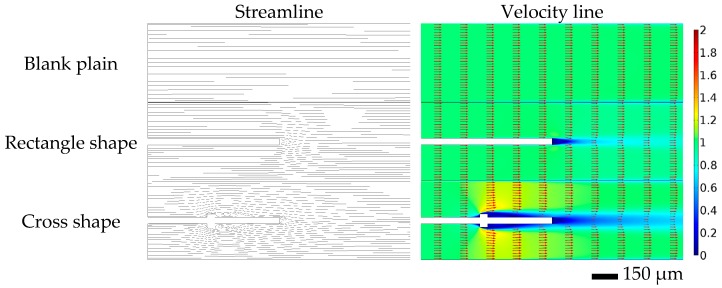
Velocity vector map (right) and corresponding streamline (left) of the plain blank microchannel, the microchannel with microstructure of the rectangle shape, and the microchannel with microstructure of the cross shape (velocity vector: red arrows; streamline: black lines).

To acquire a deep understanding of the mechanism of flow promotion, 2-dimensional vector maps and the corresponding streamlines were obtained by the FEM simulation, as shown in Figure 4. The velocity vector map is on the right and the corresponding streamline is on the left. The flow state in the plain blank channel was laminar and the streamlines remained parallel. Thus, convection in the direction transverse to the main flow was negligible and the crossover remained diffusion-limited. In the case of the rectangle shape, the streamlines converged toward the center after the fluid barrier at the end of the long strip, which caused a swirling current and perturbation [18]. The tiny swirling quickly trended toward laminar. The periodic partition walls served as guidelines rather than promoters of the laminar extraction. Additionally, compared to the rectangle shape, a slight turbulence was formed behind the bulge of the cross shape [21]. The employed auxiliary microstructures perturbed the laminar flow field. In each individual phase, transverse transport of solutes quickened, and the solute concentration changed to a relatively uniform value. The diffusion of solvent molecules from high to low concentration in the aqueous feed phase and the organic extractant phase was expedited, respectively. Thus, it maintained a relatively high concentration difference in the interface of the two phases. The mass transfer in the interface of the two phases was accelerated by the rapid movement of solvent molecules and the relatively high concentration difference. In the case of the cross shape, the extraction efficiency was improved through not only increasing transverse transport of solutes in each phase, but also promoting the mass transfer on the interface of the two phases. 

**Figure 5:**
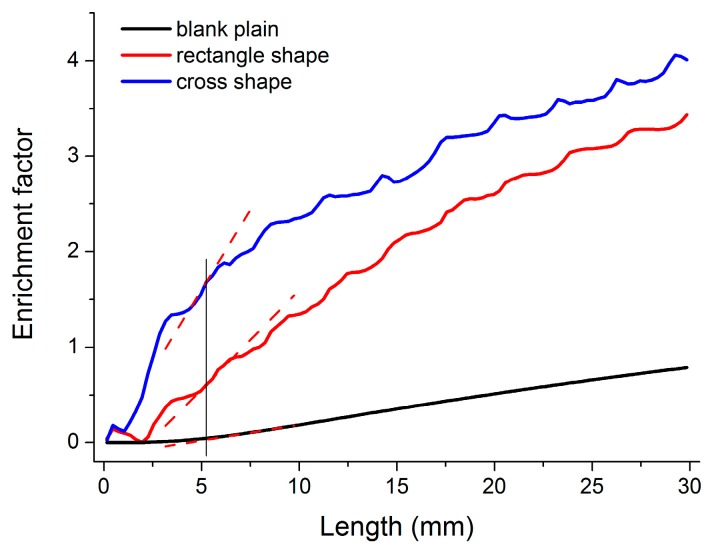
Simulation result of enrichment factor in the center of the organic extractant phase in the microfluidic system for a single extraction cycle (dashed lines: the slope of fitted curves respectively).

Figure 5 shows the simulation result of the enrichment factor in the center of the organic extractant phase for one single extraction cycle. Several significant rise and flat fluctuations appear in the curves, which correspond to the periodic auxiliary microstructures. It is assumed that the rise of the fitted curves corresponds to a fast extraction process in the interface, and the curve became flat in the division of microstructures. In the same site of the channel, the slope of the curve in the cross shape is superior to that of the rectangle shape, and the plain blank is the worst, as shown by the dashed lines in Figure 5. A large slope factor led to a high extraction efficiency of the microfluidic chip. Therefore, the microfluidic chip integrated with the auxiliary microstructure of the cross shape is assumed to be more effective in the extraction. Theoretically, increasing the slope of the rise and shortening the flat portion of the curve could enlarge the enrichment factor of the system, as well as the extraction efficiency. One important rule to design the size of the auxiliary microstructure is ensuring the recovery of the laminar flow in the microchannel. Another rule is to enhance the perturbation in the microchannels as much as possible. On account of the small Reynolds numbers far less than 1 and the guideline function of auxiliary microstructures, the laminar flow state in the microfluidic devices kept steady despite the turbulence caused by the auxiliary microstructures.

### 3.3. Performance of Microfluidic Chip

**Figure 6:**
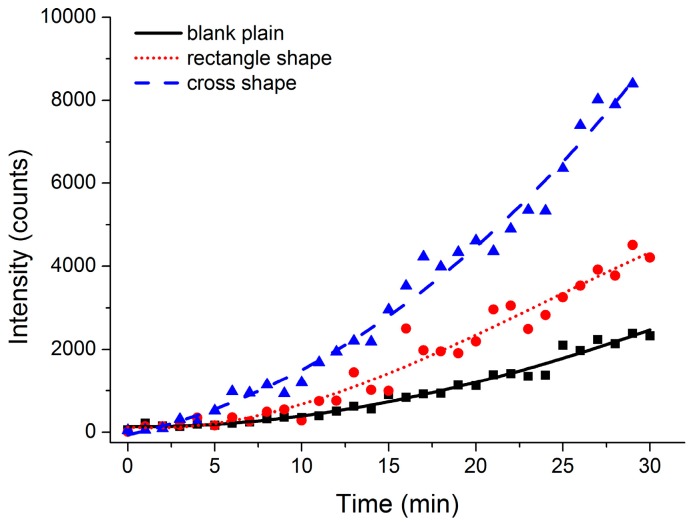
The laser-induced fluorescence intensity of the concentrated Rh6g solvent after an extraction time of half an hour. The velocity of the aqueous and organic phases was set at 2 and 12 mL·h^−1^, respectively.

The performance of the microfluidic chip was demonstrated in a darkroom. An aqueous solution of Rh6g with a concentration of 10 ppb was tested in the microfluidic system. The laser-induced fluorescence intensity of the concentrated Rh6g solvent is presented in Figure 6. A significant enrichment of the Rh6g solvent was observed in the experiment with an intense color change, first appearing transparent without the Rh6g molecule diffused and gradually darkening to bright orange with the increase of the Rh6g concentration. The velocity of the aqueous Rh6g solution was set as 2 mL·h^−1^, and the velocity of the n-octyl alcohol extraction agent was set as 12 mL·h^−1^, which was six times the aqueous velocity mentioned previously. The data results also reflected the enrichment of the concentration of the Rh6g solvent. The extraction efficiency was improved as the auxiliary structures modified the microchannel. In the case of 2 mL·h^−1^ of the aqueous rate, as shown in Figure 6, the fluorescence intensity of the rectangle shape was 190% that of the plain blank channel within the same amount of extracting duration, and the fluorescence intensity of the cross shape was almost 350% that of the plain blank channel. The auxiliary microstructure of the cross shape was more effective than the rectangle shape, and the rectangle shape was better than the plain blank channel as well. The impact of auxiliary microstructures was consistent and in good agreement with FEM simulation results, as shown in Figure 5. The mass transfer and extraction were carried out in the vicinity of the two-phase interface. The auxiliary microstructures in the center line of the microchannel served as guidelines and promoted the mixing of the reactants at the same time [19]. The diffusion of the Rh6g molecule in the aqueous and organic phases was accelerated on account of a limited turbulent flow caused by the barrier in the microchannel. The cross shape located in the center line of the channel was considered to be more effective for solvent enrichment in the laminar extraction process.

**Figure 7:**
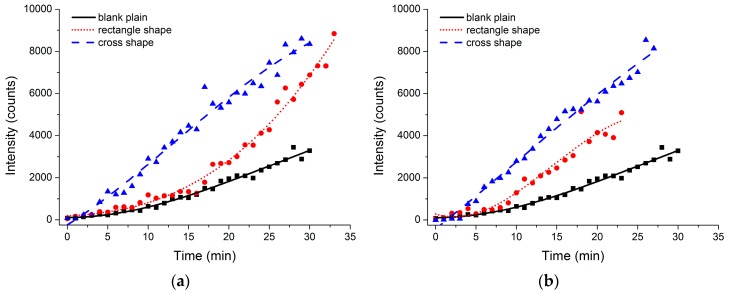
The laser-induced fluorescence intensity of the concentrated Rh6g solvent after an extraction time of half an hour. (**a**) The velocity of the aqueous and organic phases was set at 3 and 18 mL·h^−1^respectively; (**b**) the velocity of the aqueous and organic phases was set at 4 and 24 mL·h^−1^, respectively.

In order to verify the influence of the velocity of phase flow, various sampling rates were studied in the subsequent experiment, as shown in Figure 7. In detail, the aqueous velocity was set as 3 mL·h^−1^ in Figure 7a and 4 mL·h^−1^ in Figure 7b, and the organic velocity was set as 18 and 24 mL·h^−1^, respectively. For various sampling rates, the fluorescence intensity of the microfluidic chip of the cross shape was superior to that of the rectangle shape, and the rectangle shape was greater than the plain blank channel. There was no significant improvement on extraction efficiency as the sampling velocity increases. However, the laminar flow became unstable after the long-term extraction procedure, as manifested in the unsmooth fitted curve of fluorescence intensity as the sampling rate increases. Therefore, 2–12 mL·h^−1^ or lower was validated as the appropriate sampling velocity value for long-term extraction.

## 4. Conclusions

In this study, a real-time fluorescence detection microfluidic chip integrated with auxiliary microstructures was fabricated. The situation of phase interface was adjusted by the flow rates of the two phases. It is efficient to promote the limit of detection of a microfluidic chip integrated with auxiliary microstructures. The auxiliary microstructure of the cross shape was considered to be more effective in accelerating mass transfer and enlarging the enrichment factor in the laminar liquid–liquid extraction system. The microfluidic chip was proved to be stable for long-term extraction and real-time detection of trace analytes. In future work, the microfluidic system has significant application prospects in such fields as environmental monitoring and biochemical detection*.*

## References

[1] DeMello A.J. (2006). Control and detection of chemical reactions in microfluidic systems. Nature.

[2] Xiao J., Song F.C., Seo S.W. (2013). A multilayered integrated sensor for three-dimensional, micro total analysis systems. J. Micromech. Microeng..

[3] Kovarik M.L., Ornoff D.M., Melvin A.T., Dobes N.C., Wang Y.L., Dickinson A.J., Gach P.C., Shah P.K., Allbritton N.L. (2013). Micro total analysis systems: Fundamental advances and applications in the laboratory, clinic, and field. Anal. Chem..

[4] Culbertson C.T., Mickleburgh T.G., Stewart-James S.A., Sellens K.A., Pressnall M. (2014). Micro total analysis systems: Fundamental advances and biological applications. Anal. Chem..

[5] Watts P., Wiles C., Haswell S.J., Pombo-Villar E. (2002). Solution phase synthesis of β-peptides using micro reactors. Tetrahedron.

[6] Burns M.A., Johnson B.N., Brahmasandra S.N., Handique K., Webster J.R., Krishnan M., Sammarco T.S., Man P.M., Jones D., Heldsinger D. (1998). An integrated nanoliter DNA analysis device. Science.

[7] Fu A.Y., Chou H.P., Spence C., Arnold F.H., Quake S.R. (2002). An integrated microfabricated cell sorter. Anal. Chem..

[8] Anderson C.R., Rupp H.S., Wu W.H. (2005). Complexities in tetracycline analysis-chemistry, matrix extraction, cleanup, and liquid chromatography. J. Chromatogr. A.

[9] Strehle K.R., Cialla D., Rosch P., Henkel T., Kohler M., Popp J. (2007). A reproducible surface-enhanced raman spectroscopy approach. Online SERS measurements in a segmented microfluidic system. Anal. Chem..

[10] Chen L.X., Choo J.B. (2008). Recent advances in surface-enhanced raman scattering detection technology for microfluidic chips. Electrophoresis.

[11] Henderson R.K., Baker A., Murphy K.R., Hamblya A., Stuetz R.M., Khan S.J. (2009). Fluorescence as a potential monitoring tool for recycled water systems: A review. Water Res..

[12] Rezaee M., Yamini Y., Faraji M. (2010). Evolution of dispersive liquid-liquid microextraction method. J. Chromatogr. A.

[13] Brody J.P., Yager P. (1997). Diffusion-based extraction in a microfabricated device. Sens. Actuators A Phys..

[14] Fleming K.K., Longmire E.K., Hubei A. (2007). Numerical characterization of diffusion-based extraction in cell-laden flow through a microfluidic channel. J. Biomech. Eng..

[15] Huh Y.S., Jeon S.J., Lee E.Z., Park H.S., Hong W.H. (2011). Microfluidic extraction using two phase laminar flow for chemical and biological applications. Korean J. Chem. Eng..

[16] Lin B. (2011). Microfluidics: Technologies and Applications.

[17] Xiao H., Liang D., Liu G., Guo M., Xing W., Cheng J. (2006). Initial study of two-phase laminar flow extraction chip for sample preparation for gas chromatography. Lab Chip.

[18] Maruyama T., Kaji T., Ohkawa T., Sotowa K.-I., Matsushita H., Kubota F., Kamiya N., Kusakabe K., Goto M. (2004). Intermittent partition walls promote solvent extraction of metal ions in a microfluidic device. Analyst.

[19] Marschewski J., Jung S., Ruch P., Prasad N., Mazzotti S., Michel B., Poulikakos D. (2015). Mixing with herringbone-inspired microstructures: Overcoming the diffusion limit in co-laminar microfluidic devices. Lab Chip.

[20] Tagawa T., Aljbour S., Matouq M., Yamada H. (2007). Micro-channel reactor with guideline structure for organic–aqueous binary system. Chem. Eng. Sci..

[21] Iaccarino G., Ooi A., Durbin P.A., Behnia M. (2003). Reynolds averaged simulation of unsteady separated flow. Int. J. Heat Fluid Flow.

[22] Friend J., Yeo L. (2010). Fabrication of microfluidic devices using polydimethylsiloxane. Biomicrofluidics.

[23] Zhou Y. (2013). A Fluorescence Oil Detection Device.

[24] Huang Y.S., Meng T., Guo T., Li W., Yan W.L., Li X.R., Wang S., Tong Z.P. (2014). Aqueous two-phase extraction for bovine serum albumin (BSA) with co-laminar flow in a simple coaxial capillary microfluidic device. Microfluid. Nanofluid..

